# Use of Focus Groups to Identify Food Safety Risks for Older Adults in the U.S.

**DOI:** 10.3390/foods11010037

**Published:** 2021-12-24

**Authors:** Melissa Kavanaugh, Kathleen Fisher, Jennifer J. Quinlan

**Affiliations:** 1 Department of Nutrition Sciences, College of Nursing and Health Professions, Drexel University, Philadelphia, PA 19102, USA; mmk97@drexel.edu; 2 Graduate Nursing, College of Nursing and Health Professions, Drexel University, Philadelphia, PA 19102, USA; kmf43@drexel.edu

**Keywords:** older adults, food safety, focus groups, consumer education, Health Belief Model, listeria, poultry, behavioral theory

## Abstract

Older adults are vulnerable to foodborne illness; however, many do not follow safe food handling guidelines that would reduce their risk of infection. Virtual focus groups were used to explore older adults’ food handling and consumption practices and to understand how to apply the Health Belief Model for food safety research with respect to older adults. Thirty-nine adults between the ages of 56 and 80 participated in the study. Most participants reported eating poultry and eggs, whereas few reported eating precut fruit or raw sprouts. The majority were not using a cooking thermometer for all types of poultry and did report washing raw poultry. Participants were generally resistant to the idea of heating deli meats. Most focus group participants did not perceive themselves as being personally susceptible to foodborne illness. They did, however, express food safety concerns related to specific foods, such as melons and bagged salads, and they reported taking precautions to limit health risks from these foods. Regarding the Health Belief Model, our results indicate that the construct of perceived susceptibility could be expanded to include perceived risk, which refers to an individual’s belief about the likelihood that a food might be contaminated with a foodborne pathogen. These results should be confirmed among a nationally representative sample of older adults.

## 1. Introduction

Across many industrialized nations, *Campylobacter* and *Salmonella* are leading causes of foodborne illness, whereas *Listeria* and *Salmonella* are leading causes of foodborne illness-related mortality [1,2,3,4,5]. Individuals of all ages are at risk of contracting foodborne illnesses; however, certain segments of the population are more vulnerable than others. Older adults may be more likely to present with comorbid conditions such as cancer or gastritis, or they may be taking certain medications that have the potential to increase their susceptibility to foodborne illness [6]. Older adults are among the groups most susceptible to foodborne illness, and once they become infected, they generally experience higher rates of hospitalization and mortality [6,7,8,9].

In the United States, it is estimated that *Salmonella*, *Campylobacter* and *Listeria* account for more than two hundred and twenty thousand illnesses among adults who are 65 years and older each year [8]. In the U.S. as well as in the European Union, *Listeria* is responsible for fewer infections than either *Salmonella* or *Campylobacter*; however, even though the absolute number of infections is lower, older adults account for the majority of cases [8,10]. Once older adults are infected by *Listeria*, they are also more likely to present with severe complications, such as septicemia, and they are more likely to die from them [8,11]. Older adults may also experience more severe consequences with respect to infection from *Salmonella*. In Canada, between 2000 and 2010, 50% of non-typhoidal *Salmonella*-related hospitalizations and 82% of associated deaths occurred in seniors [12]. In the U.S., adults who are 65 years of age and older are also the most likely to be hospitalized or die as a result of salmonellosis [8]. Conversely, in the United States, older adults are less likely to be infected by *Campylobacter*, but they are still more likely than any other age group to be hospitalized or die as a result of campylobacteriosis [8]. Infection by any of these pathogens represents a significant health concern for older adults and this underscores the need to prevent these foodborne illnesses. Additionally, the population of older adults is increasing in many westernized countries, including the United States, United Kingdom and parts of the EU, making this increased susceptibility a growing risk regarding increased foodborne illnesses overall [13,14,15,16,17].

Despite their vulnerability, older adults have reported a number of unsafe food consumption and food-handling practices, including consuming undercooked eggs, failing to use a cooking thermometer and mishandling of ready-to-eat (RTE) foods [18,19,20,21]. It was previously recognized that older adults were more observant of food safety recommendations when compared to younger age groups [22,23]; however, the research studies positing an association between age and adherence are fifteen to twenty years old. The participants included in more recent investigations likely represent a newer generation of older adults with distinct food handling patterns. In addition to unsafe food-handling practices, the overwhelming majority of older adults (88%) mistakenly believe that their risk of foodborne illness is low [24]. Therefore, effective education interventions are needed to correct misconceptions and incentivize this new generation of older adults to follow safe food-handling guidelines.

Experts recommend that future food safety education interventions are informed by a theory of behavior change [25,26] because these theories provide a structured framework offering insight into why an individual or a group of individuals would modify their behavior [27]. However, there is a recognition that, in the context of food safety, existing theories might not adequately explain the relationships between psychosocial constructs and behavior among certain subgroups of the population, including older adults [26]. Therefore, experts also advocate employing formative research, such as qualitative focus groups or interviews [25,26], in conjunction with a theory of behavioral change so that the chosen theory can be contextualized to fit the target population.

The Health Belief Model (HBM) is an established theory of behavioral change, which has been used in practice for over 70 years, to explain a wide range of health-related behaviors [28]. Briefly, the HBM states that an individual will take a health-related action if they believe that they are susceptible to the related illness, if they believe that the illness will be severe, if they perceive a benefit from taking action, and if they perceive that the expected benefit outweighs any perceived barrier(s) [28]. According to the HBM, individuals may also take a health-related action if they receive external cues to action, such as educational cues or media messages [28]. The HBM appears promising for use with older adults because several of its constructs, such as cues to action, may be applicable to what is known about older adults in the context of food safety [29]. However, only one food safety study targeting older adults employed the HBM [29]. Therefore, formative research is needed to fully elucidate how the HBM may apply to older adults’ food-handling practices. The objective of the research presented here was to use qualitative focus groups in order to understand how to use the HBM with older adults in the context of food safety. We also used the qualitative focus groups to determine participants’ food handling and consumption practices in connection with foods that could be contaminated with *Salmonella*, *Campylobacter* or *Listeria*.

## 2. Materials and Methods

### 2.1. Focus Group Questioning Route

Drexel University’s Institutional Review Board approved the study protocol. The focus group questioning route (Questionnaire S1) was developed to determine the frequency of consumption of foods that could be contaminated with *Salmonella*, *Campylobacter* or *Listeria,* as well as related food-handling practices (Table 1). The Health Belief Model also served as a framework to guide the development of focus group questions, to capture behavioral constructs that could be related to respondents’ food-handling behaviors. For example, the focus group moderator asked questions such as: (1) what are your food safety concerns related to eating runny eggs? (perceived threat); (2) does it surprise you that, as a group, older adults are more likely to be hospitalized with foodborne illness? (perceived threat); (3) the USDA recommends using a cooking thermometer for all types of poultry. What would it take for you to use a cooking thermometer for all types of poultry? (perceived barriers); (4) how do you get information about safe food handling? (cues to action). The focus group questioning route featured semi-structured questions that guided the discussion but allowed participants to provide open-ended responses. This format was utilized to provide a balance between the dependability, credibility and confirmability of qualitative research results [30]. At the end of each section, the moderator reiterated the main points and asked participants to confirm or amend the moderator’s impressions. Prior to conducting the focus groups with older adults, the questioning route was pilot-tested with four graduate students from Drexel University to confirm the clarity of questions, the anticipated length of the focus group session, and the feasibility of the focus group format, which would be conducted using the Zoom video-conferencing platform [31].

### 2.2. Recruiting Procedures

ResearchMatch© was used to recruit eligible study participants. ResearchMatch© offers a free service that connects researchers from participating institutions with study respondents who have previously registered with its platform [32]. Respondents were eligible to participate if they were 55 years of age or older, lived independently, prepared at least five meals per week, had reliable Internet access, owned a device with a working camera that would allow the participant to attend an online meeting and who were comfortable being audio- and video-recorded. Participants were excluded if they did not have Internet access and/or if they were unable to read and understand English. A combination of convenience and purposive sampling [33] was used to recruit participants. Specifically, to ensure the credibility and transferability of research results, the composition of the sample was assessed mid-way through recruiting, and subsequent recruiting was conducted to ensure that the final participant sample was diverse [30]. A preapproved message was sent via ResearchMatch© to individuals, 55 years of age and older, who were registered with its service. Interested respondents were contacted by the research team via phone. Informed consent was obtained from 42 respondents.

### 2.3. Focus Group Administration

Participants were contacted both the day before and the morning of their focus group session to confirm their attendance. Login information was not sent until one hour prior to the start of the focus group session, as is consistent with recommendations made by Tuttas (2015) for focus groups conducted in the virtual environment [34]. Focus groups were conducted until theoretical saturation was achieved, meaning that participants were not expressing new ideas [35]. Six focus groups were conducted in total, each lasting approximately 90 min. All focus groups were conducted using the HIPPA-compliant version of Zoom. All sessions were audio- and video-recorded using Zoom. The first author led all focus group sessions, and a second researcher took notes and assisted with focus group administration. Focus groups consisted of between five and eight individuals.

### 2.4. Data Analysis

Focus group recordings were first transcribed verbatim using the Microsoft Word© transcription feature. The first author subsequently listened to the recordings alongside the transcript to ensure that Microsoft Word© accurately captured the focus group responses. Next, the first author manually coded each transcript according to codebook definitions that were developed a priori in order to capture foods consumed and food-handling practices, as well as the Health Belief Model constructs: perceived threat, perceived barriers and cues to action. In vivo codes were also created for unanticipated behavioral constructs or food-handling practices, and magnitude coding was used to highlight the frequency of key codes [36]. Finally, similar codes were grouped together to form categories in an iterative process that ultimately identified the main theme and related subthemes [36]. Variations in the data, as well as contradictory responses, were also recorded. After the completion of manual coding, the transcripts were uploaded to NVivo qualitative analysis software [37], and the transcripts were coded a second time in order to confirm the results of the manual analysis.

To ensure credibility and trustworthiness, the focus group moderator composed a memo to document initial impressions compared to expectations, following each focus group session. Memos were also written throughout data analysis to document how codes and themes evolved and how consensus was ultimately reached [38]. Two researchers analyzed two of the six transcripts and reached a consensus with respect to the prominent concepts and themes embodied by those two transcripts. Then, one researcher analyzed the remaining transcripts and provided a rationale with support from the subsequently analyzed transcripts for any changes made to the prominent themes previously established. Once a consensus was reached regarding the major themes and subthemes related to all six transcripts, a separate researcher, experienced in qualitative inquiry, performed an audit of the qualitative analysis protocol [38]. The audit included a review of example memos from each stage of the research process, a review of the a priori codebook and coding procedure, and descriptions of how codes were combined to arrive at themes, as well as the process of reaching consensus. As a result of this audit, it was determined that conclusions were reached in a systematic and reproducible manner.

## 3. Results

### 3.1. Participant Characteristics

Thirty-nine respondents participated in the focus groups. Sixty-four percent of the participants were female. Participants ranged from 56 to 80 years of age; the majority were in their 60s (59%) or 70s (26%). Of the participants, 72% were Caucasian, 15% were African American, and 10% were Hispanic or Latino. Participants lived throughout the United States, with 34%, 31%, 31% and 14% living in the South, West, North-East and Mid-West, respectively.

### 3.2. Food Consumption and Handling Patterns

#### 3.2.1. Poultry and Eggs

Most participants reported eating eggs, and approximately half of the participants reported eating raw or runny eggs. Over-easy eggs were most commonly cited as the type of runny egg consumed; however, some participants also reported consuming other types of runny eggs, as well as raw eggs in foods such as raw batter or eggnog. All but two participants reported that they always stored their eggs in the refrigerator.

All participants, with the exception of one, ate some form of poultry (whole, parts or ground). Most seemed to know the recommended methods for thawing poultry (in the refrigerator, microwave, or cold water). Less than one-quarter reported thawing poultry at room temperature. Participants were generally aware of practices to avoid cross-contamination, particularly with respect to raw poultry (Table 2). Approximately one-third of participants washed raw poultry, and many of those who did so seemed to be unaware of the recommendation not to do so. Several participants self-identified as not washing poultry; however, they “rinsed” their poultry, and did not recognize that rinsing and washing pose similar food safety risks (Table 2). The majority of participants were not using a thermometer at all, or they were using it only for unfamiliar dishes or for whole poultry. Generally, participants used sight, duration, and experience to determine the doneness of poultry (Table 2).

#### 3.2.2. Produce

Only about one-quarter of the participants reported eating raw sprouts that were purchased at the grocery store or at restaurants, but for most who were consuming raw sprouts, they were an incidental, rather than a regular component of participants’ diets (Table 3). Several participants also reported that they used to eat sprouts but stopped due to food safety concerns. More than half of the participants reported eating bagged salad products; the remaining participants either purchased and consumed full heads of lettuce or they avoided leafy greens altogether. Health and convenience were cited as factors in the use of bagged or boxed salad products. Some participants also reported avoiding bagged and boxed salads due to food safety concerns. The majority of participants reported consuming melon that was purchased whole, whereas only about one-sixth of participants reported consuming pre-cut melon. Many participants knew that the outside of melons should be washed in order to avoid transferring germs from the rind to the flesh of the fruit; however, a few participants did not understand the logic with respect to washing melons (Table 3).

#### 3.2.3. Ready-to-Eat Foods

The majority of participants were eating deli salads; however, this included both homemade deli salads and those purchased from a retail establishment such as a grocery store or restaurant. In contrast, only a little over a third of the participants reported ever consuming deli salads from a retail establishment. Many participants who did consume deli salads preferred to make their own for reasons of taste preference, and several participants expressed food safety concerns with respect to retail deli salads (Table 4). Just under half of the participants reported eating deli meats, at least on occasion. Participants who reported avoiding deli meats were generally doing so for reasons of health (i.e., high sodium content). Some participants reported keeping opened packages of deli meats for longer than 5 days, whereas other participants reported throwing out opened packages of deli meat within 5 days. Participants were generally resistant to the idea of heating deli meats; however, a few were receptive (Table 4). Others were more receptive to avoiding deli meats altogether when faced with the recommendation to heat deli meats (Table 4). The majority of participants also reported eating hot dogs; however, for most, it was an occasional food consumed several times per year. Most participants reported throwing opened packages of hot dogs away within one week, and most also reported cooking hot dogs. However, several participants reported eating cold hot dogs. Most participants did not own a refrigerator thermometer.

### 3.3. Health Belief Model Constructs

#### 3.3.1. Perceived Threat

Most participants did not agree that they were more susceptible to foodborne illness due to their age. Moreover, if they did agree that older adults, as a group, could be more susceptible, they generally did not recognize themselves as being personally susceptible (Table 5). In contrast, participants who had previous experience with severe foodborne illness reported modifying their behavior accordingly (Table 5). Participants did express perceived food safety risks related to specific foods, and they did take precautions to mitigate those perceived risks. Participants were most concerned about poultry, raw sprouts, bagged or boxed salad products, and melons (Table 5). There was generally a lack of concern related to eating raw or runny eggs; however, some older adults were concerned that eggs were fully cooked, and others were receptive to changing their behavior, once they were informed that raw and runny eggs present a food safety risk (Table 5).

#### 3.3.2. Cues to Action

The relationship between recalls or outbreaks and changes in food safety behavior was mentioned directly or alluded to in four of the six focus groups (Table 6). The foods that were mentioned in connection with recalls and/or outbreaks were poultry, sprouts, bagged or boxed salad products, and melons. These were also the foods that participants consistently mentioned as being risky. Previous food safety experience, such as working as a cook, and its impact on current food-handling practices were mentioned in five out of the six focus groups (Table 6).

#### 3.3.3. Perceived Barriers

Participants reported engaging in unsafe food-handling practices because they were habituated to the practice or because previous experience served to reinforce their practices (Table 7). Habit and experience were particularly powerful barriers with respect to eating raw or runny eggs, cooking without a thermometer, and washing poultry. Participants also reported engaging in unsafe food-handling practices for reasons of convenience. Convenience was cited as the reason for thawing poultry on the counter and for consuming bagged or boxed salad products. Participants’ food choices were largely guided by their taste preference, and this could result in both safe and unsafe practices. For example, some participants preferred runny eggs, whereas others avoided retail deli salads for reasons of taste preference. Finally, the family was mentioned as a source of food safety information in 4 of the 6 focus groups, and advice given by family members was often incorrect (Table 7).

#### 3.3.4. Other Results

The focus groups revealed that participants had misconceptions about food safety risks. The most common misconception was that purchasing foods from a “reputable source” was a reliable method to reduce the risk of foodborne illness. Health as a factor in food choice came up in all six of the groups. Seeking out fresh high-quality food could result in both safe and unsafe food-handling practices. For example, many participants reported avoiding deli meats due to the sodium content, but participants also reported eating bagged or boxed salad products as a way to make sure that they were meeting their daily requirements for vegetables. Concern over other people’s germs came up in 4 of the 6 focus groups. One participant related, “*Other people’s hands have been on that meat, so I got to wash it*”. The presence or absence of others in the household is another factor that could have a positive or negative impact on food safety behaviors. Older adults who live alone may be less concerned with respect to their vulnerability or they may not throw away perishable foods within prescribed time frames. One participant who lived alone said, “*And at my age. You know, if I’m enjoying some food and that kills me, you know, so be it. I’m single, I don’t have kids, I don’t have family nearby*”. However, some participants related that they consumed potentially risky foods, such as bagged salads, because someone else in the household purchased the item.

Older adults also seemed to have sufficient self-efficacy (another Health Belief Model construct). Based on our focus group conversations, most older adults seemed to believe in their own abilities; however, the challenge may be in convincing them that what they’ve always done is not correct. As one participant very succinctly stated, “*I’ve cooked long enough that I generally can tell by looking at it*” [that it is cooked]. With respect to knowledge, many participants were aware of *Salmonella*. They knew that it was a bacterium of concern with respect to poultry and some also knew that *Salmonella* could contaminate eggs. Fewer participants had heard of *Listeria* but, when prompted, some participants knew that *Listeria* could contaminate melons, sprouts and greens. Most participants were not aware that *Listeria* could contaminate ready-to-eat foods such as deli meats and hot dogs. Finally, only a few participants had heard of *Campylobacter*.

## 4. Discussion

It was previously recognized that older adults were more observant of food safety recommendations when compared to younger age groups [22,23]; however, the results of more recent research suggest that, regardless of whether they are more or less adherent to the recommendations than younger consumers, older adults engage in numerous unsafe food-handling practices [18,19,20,21,39,40,41]. With the exception of poultry-washing, the risky food-handling behaviors expressed by focus group participants were consistent with those reported as part of more recent research and included behaviors such as a failure to use a cooking thermometer or the consumption of undercooked eggs [18,19,21,39]. Previous quantitative surveys of both older and younger consumers reported that approximately 70% of them wash raw poultry at least some of the time [21,41,42]. Only about one-third of focus-group participants reported washing raw poultry. Results from focus group research, however, are not meant to be generalizable to an entire population [35].

Based on the focus group responses, it appears that older adults were eating both poultry and eggs regularly, whereas the high-risk produce and ready-to-eat food (RTE) items included in the questioning route were consumed with varying frequency. Traditionally, food safety education interventions have included many food safety behaviors [43,44,45,46]; however, there is evidence that education interventions targeting older adults are most effective when they are limited to one or two simple messages [47]. Therefore, it may be advantageous to focus future interventions on practices related to foods that comprise a substantial portion of older adults’ diets. Few food-safety research studies have sought to characterize how often high-risk foods are consumed [48,49]. This gap in the literature could be addressed by including food frequency questions within a food safety survey.

Another goal of this research was to understand how to use the Health Belief Model (HBM) in terms of older adults. After analyzing focus group discussions, the definitions of perceived barriers and cues to action appear applicable to older adults’ food handling behaviors. The HBM defines perceived barriers as the perceived costs associated with taking a health-related action [28], whereas cues to action are identified as the educational cues and media messages, such as brochures or news stories, respectively, that may prompt an individual to adopt a health-related behavior [28]. The perceived barriers of taste preference, habit/experience, convenience, and family influence were the most prominently expressed and seemed to explain certain unsafe food handling behaviors among participants. Young and Waddell (2016) noted similar barriers as part of their systematic review of qualitative food safety research studies [50]. Conversely, cues to action, especially recall and outbreak information, appeared to explain some safe food-handling practices, such as washing whole melons. Hanson and Benedict (2002) also reported significant associations between older adults’ use of safe food-handling practices and their recognition of both educational cues and media messages [29]. Thematic analysis of the focus group responses suggests that including recall information in a food safety intervention may incentivize safe handling of the recalled food; however, this proposed relationship should be confirmed among a representative sample of older U.S. adults.

It appears that the HBM could serve as a framework to guide future intervention programing for older adults; however, when the HBM is used with older adults for food safety research, we propose modifying the application of the perceived threat construct. Under the HBM, perceived threat encompasses both perceived susceptibility and perceived severity. Perceived susceptibility refers to an individual’s belief about the likelihood of contracting an illness, whereas perceived severity refers to an individual’s belief about how sick they might become if they contract the illness [28]. The only food safety research study to characterize the association between perceived threat and food handling behaviors in older adults reported a significant association between some safe food-handling practices and perceived severity but no association between the former and perceived susceptibility [29]. The relationship between perceived severity and safe food-handling practices that was reported by Hanson and Benedict (2002) is consistent with the thematic analysis of the focus group responses, suggesting that perceived severity may explain some safe food-handling practices, whereas the lack of association between perceived susceptibility and food-handling practices reported by Hanson and Benedict (2002) could be related to the way that their questions were phrased. The survey asked participants to agree or disagree with general statements of perceived susceptibility, such as “I doubt I will ever get a foodborne illness” [29,51] (p. 52). Our focus group results indicated that older adults may not perceive a personal susceptibility to foodborne illness; this interpretation is supported by the existing literature [24,52]. In contrast, older adults in the focus groups did perceive risks related to specific foods, and they reported taking preventative measures based upon their risk perception. Therefore, we suggest that when the HBM is used with older adults for food safety research, measures of perceived susceptibility could be expanded to include *perceived risk*, which refers to an individual’s belief about the likelihood that a food might be contaminated with a foodborne pathogen that could cause a foodborne illness.

Our results with respect to food-handling practices were generally consistent with those of recent research studies, finding that older adults do not adhere to a number of food safety guidelines. Unique to this research, however, is an identification of the possibility that older adults may not frequently consume risky RTE foods due to both health and quality concerns. Collectively, these results suggest the need for future food safety education interventions targeting older adults; however, these research results should be considered in the context of several limitations. The sample size used for this research was small and was not representative of the U.S. population of older adults. For example, unrepresented groups, such as Asian American consumers, might have different food-handling practices. Moreover, in order to reduce the participant burden, we did not capture certain demographic factors, such as education level, which might be associated with food safety practices. The results of focus group research are not meant to be generalized to the target population. Instead, focus groups are a form of qualitative inquiry, the purpose of which is to explore a topic and generate ideas or hypotheses, in order to inform the development of future quantitative research studies.

The results of this research can be used to inform the development and validation of a food safety survey to be administered to a representative sample of older adults. We recommend including a food frequency-type questionnaire in the survey in order to determine how often high-risk foods are consumed. In addition, it appears that the Health Belief Model could serve as a framework to guide future food safety education intervention programming for older adults; however, we suggest that the HBM is also incorporated into future quantitative studies in order to further elucidate the relationships between HBM constructs and safe food-handling practices among older adults. Ultimately, quantitative research among a nationally representative sample of older adults is needed to confirm the results of this qualitative analysis, as well as to make concrete recommendations for consumer education. 

## Figures and Tables

**Table 1:** Common foods that could be contaminated with *Salmonella*, *Campylobacter* or *Listeria,* and related food-handling practices included in the focus group questioning route.

Foods		Handling Practices	
Eggs	Melons	Cooking eggs	Storage of melons
Poultry	Deli meats	Washing poultry	Storage of deli meats
Sprouts	Deli salads	Cooking thermometer use	Heating of deli meats
Bagged salads	Hot dogs	Thawing poultry	Purchase of retail deli salads
		Use of raw sprouts	Storage of hot dogs
		Use of bagged salads	Heating of hot dogs
		Washing melons	Refrigerator thermometer use

**Table 2:** Food handling behaviors related to poultry, with supporting quotes from the focus group discussions.

Related Behavior	Example
Prevention of cross-contamination	Like [he] said, clean my hands before, I don’t cross. I clean my knives and I clean the cutting board if I don’t have another cutting board. So with hot water and soap, I rinse it before I chop anything else. Female, Caucasian
Washing poultry	I actually thought you were supposed to rinse it, and I guess I got the wrong message or didn’t get the other message. Male, Caucasian
	I used to wash poultry, but then now we, they recommend that we don’t do it because if there is bacteria, we could spread it. So I just rinse it quickly to get the, you know, the juices that are off, at least, off of it. Female, Caucasian
Cooking thermometer	I use a cooking thermometer depending on what type of meat it is. Um, like, uh, recently over the holidays, baked a duck, so I use it, ‘cause it cooks differently than a chicken would. Female, African American
	I only use a thermometer when I’m cooking a turkey. Female, African American
	I don’t use it [a thermometer] with chicken usually because we piece it before we cook it. Male, Caucasian
	After 20 min, boiling, frying or whatever, it’s gonna be done. Female, Hispanic

**Table 3:** Food handling behaviors related to produce, with supporting quotes from the focus group discussions.

Related Behavior	Example
Consumption of raw sprouts	I guess sometimes you get a sandwich that has sprouts on it or a wrap, if it comes in a salad. I just eat those, I don’t really give it much thought. Male, Caucasian
Washing melon	Yeah, I’ve never washed melon in my life, so I, are you talking about the outside? […] I don’t eat the outside. I’m eating the inside. Female, African American, Mixed-race

**Table 4:** Food handling behaviors related to ready-to-eat foods, with supporting quotes from the focus group discussions.

Related Behavior	Example
Purchasing retail deli salads	If I ever found one I liked better than my own I might get it, but I find them woefully inadequate. Female, Caucasian
	I’ve stopped in the last couple of weeks or so because I’m again thinking that you know this may not be prepared as safely as I would like for it to be. Female, African American
Heating deli meats	A hot sliced ham from the deli does not sound appetizing. Female, Caucasian
	And I’ll have to say I mean, for me to follow that recommendation [heating deli meats] the outcome would have to be something like “asteroid heads toward Earth”. Otherwise, I’m skipping it. Male, Caucasian
	I mean, I never even thought about cooking it. That that’s something I would do. Female, Caucasian
	If I had to heat up my deli meat, I wouldn’t buy it. Female, African American

**Table 5:** Supporting quotes from thematic analysis of the focus group discussions related to the Health Belief Model construct, perceived threat.

Theme	Sub-Theme	Example
Lack of personal susceptibility, but perceived threat related to specific foods.	Lack of personal susceptibility	I don’t think so [that older adults are more susceptible to foodborne illness], because you build up immunity to that kind of stuff too as you get older. Female, Caucasian
		I think we could be more susceptible, if we’re not taking care of ourselves. Female, Caucasian
		I think it depends on the age of [the] elderly, really. Female, Mexican and Native American
		Not really, I haven’t really felt any changes [in immune response]. Male, Caucasian
	Recognition of threat may be due to past experience	I never went to the doctor and it’s never been severe enough to where I didn’t think that oh once I got it out of my system everything would be okay. Male, Asian Indian
		I was hospitalized once from *Salmonella* […] so I am super sensitive to meats, chicken, anything [….] if it touches that meat, it gets washed in bleach […] so I’m super sensitive about meats. Female, Caucasian
	Food specific concerns	Well, every time I have, you know, come across an article of some type, most of the times it’s dealt with a poultry-based meal or poultry-based meat […] I’m hyper-vigilant about even the poultry that I fix, making sure that it’s done. Male, Caucasian
		I have, yes, absolutely, specifically with sprouts because there have you know that there have been, I think there have been incidences. You know where […] sprouts sold in the supermarket have had, have been contaminated. Female, Hispanic, Mixed-race
		I tend to only buy the ones with, like, the baby leaves [bagged salads] as opposed to one that’s been chopped up because it’s less processed and less risk. Female, Caucasian
		I always assumed that was enough heat that would sterilize any bacteria concerns in the, in the yolk. [when preparing runny eggs] Male, Caucasian
		People have eaten eggs for 50 years like that [runny]. Female, Caucasian
		I do worry at times if I’ve cooked them enough. I only like runny eggs if I had some really good toast. Otherwise, I don’t usually do it. I’m concerned. Male, Caucasian
		Yeah, I’ll probably go to scrambled [after learning that the risk associated with eggs has changed since she first learned to cook]. Female, Caucasian

**Table 6:** Supporting quotes from a thematic analysis of the focus group discussions related to the Health Belief Model construct, cues to action.

Theme	Sub-Theme	Example
Cues to action that could be related to current food safety behaviors	Recalls/Outbreaks	I absolutely will not touch ground poultry products. And partly because I read about recalls all the time. Female, Caucasian
		I know a few years ago there were a lot of major, I don’t even know whether it was *Listeria*, whatever was the issue with sprouts. And they were taking them off […] so I haven’t, I don’t think I’ve had sprouts in, oh God, at least 3 or 4 years. Male, Caucasian
		I’ve tried to stay away from them [bagged/boxed salads] because of recalls on them. Male, Caucasian
		A few years ago there was a *Listeria* outbreak with the Rocky Ford [cantaloupe], killed some people […] We wash it with soap, wash it well. Male, Caucasian
	Previous food service experience	I mean, my very first job, as I mentioned earlier, was at a restaurant and seeing some of the health things there when I was 16. I’m, it’s had a big impression on me as far as food handling and food preparation and all sorts of things like that. Male, Caucasian

**Table 7:** Supporting quotes from a thematic analysis of the focus group discussions related to the Health Belief Model construct, perceived barriers.

Theme	Sub-Theme	Example
Perceived Barriers	Habit/Experience	I’ve always washed my meat […] that’s just the way I was raised. Female, African American
		I have to say if there was a danger with cake batter, my kids would have been gone a long time ago. Male, Caucasian
	Convenience	Yes, at this point in life, it’s really easy to just pour salad and you’re ready to go. Male, Hispanic
		I like to thaw it in the refrigerator but in a pinch, I have been known to take it out [for] 2 h and just let it sit there [on the counter]. Female, Caucasian
	Taste Preference	I mean I don’t really like eggs other than, you know, over easy. Female, Mexican, Native American
		I agree with him, potato salad, things like that in grocery stores is just horrible. Female, Caucasian
	Family	Grandma taught me so everything […] we’ve learned by instruction too, all right, look at the meat, gauge the meat or wash the meat. Male, African American

## Data Availability

The data presented in this study are available on request from the corresponding author. The data are not publicly available due to participant privacy.

## Supplementary Materials

The following are available online at https://www.mdpi.com/article/10.3390/foods11010037/s1, Questionnaire S1: Focus Group Questioning Route.
