# Supplementary material for: Use of Focus Groups to Identify Food Safety Risks for Older Adults in the U.S
Source: Foods. 2021 Dec 24;11(1):37. doi: 10.3390/foods11010037 (PMC8749994; doi:10.3390/foods11010037)
Supplement: Supplementary file 1 [file foods-11-00037-s001.zip › foods-1491677-supplementary.pdf]

Opening:

First Name, Favorite Food
To start us off, I am going to call on you based on where you appear on my screen. Please tell us your first name and your favorite food to prepare at home. (5 minutes)

Caregiver Status:
Does anyone in the group serve as a caregiver and prepare food for an older adult?

Eggs: Next we will discuss egg preparation and storage…(10 minutes)

1. How do you like to prepare your eggs?
2. What are your food safety concerns related to eating runny eggs? What bacteria are you concerned about?
3. The USDA recommends only eating well done eggs, does that persuade you? Or do you just love your runny eggs?
4. What other dishes do you make with eggs? Does anyone eat batter? What are your food safety concerns?
5. Where to you store your eggs?

SUMMARIZE

Poultry: Next, we'd like to talk about poultry. (15 minutes)

1. Without discussing a specific dish, from start to finish, how do you prepare raw poultry?
    a. Does anyone wash poultry?
    b. How do you thaw poultry?
    c. Do you use a cooking thermometer? What temp?
    d. Do you do anything different depending on type/cut of poultry?
2. What are your food safety concerns related to poultry? Which bacteria are you concerned about?
3. The USDA recommends using a cooking thermometer for all types of poultry, what would it take for you to use a cooking thermometer for all types of poultry?
4. The USDA recommends not washing raw poultry, does that persuade you? Or do you feel better washing it? Have any of you previously heard the recommendation not to wash raw poultry? Have you heard and decided to do it anyway?

SUMMARIZE

Ready to Eat Foods: next we will talk about foods that you can buy at the grocery store or at a convenience store that you might eat straight away or may not heat up before eating. (20 minutes)

1. Do you eat raw or lightly cooked sprouts/where do you get them?
    a. What are your food safety concerns related to raw or lightly cooked sprouts?
2. Do you eat boxed or bagged salads?
    a. What are your food safety concerns related to boxed or bagged salads?
3. Do you enjoy cold deli salads? Such as coleslaw or potato salad?
    a. Purchase or make at home?
    b. What are your food safety concerns related to cold deli salads?
4. How do you purchase and store melons such as cantaloupe or honeydew?
    a. Whole or precut?
    b. Do you wash the outside prior to cutting?
    c. How long are you keeping them?
    d. What are your food safety concerns related to melons?
5. Hot dogs - do you enjoy hot dogs? How prepared?
    a. How do you know if they are done? Do you ever eat them cold?
    b. How long do you keep them once packaged is opened?
    c. What are your food safety concerns related to hot dogs?
6. Deli Meats - do you eat deli meats such as turkey or ham?
    a. Do you buy it packaged or get it sliced at the deli counter?
    b. How long do you keep fresh sliced or packaged once the package is opened?
    c. What are your food safety concerns related to deli meats?
    d. The USDA recommends reheating deli meats to steaming hot (165F) and throwing out any opened packages of uneaten lunchmeat within 3-5 days. How do you feel about following (and/or what do you think about) those recommendations?

SUMMARIZE

Refrigeration: Next, we are going to discuss how you store foods in your refrigerator. (10 minutes)

1. Does your refrigerator have a thermometer? If so, how often do you check it? What temperature do you keep your fridge at?
2. Ask if there is time - how often do you clean out your refrigerator?

SUMMARIZE

Focus Group Closing:
The purpose of this focus group is to find out what types of foods older adults typically eat as well as how those foods are prepared in order to develop public health messaging to prevent foodborne illness. Our final questions will focus on what you think about foodborne illness and food safety. (15 minutes)

Foodborne Illness/Safety

1. Do you think that as a group older adults are more likely to get foodborne illness than others? What factors do you think contribute to this? At what age do you think older adults are more likely to get foodborne illness?
2. Does it surprise you that, as a group, older adults are more likely to be hospitalized with foodborne illness?
3. What foodborne pathogens are you concerned about? (Bring up *Listeria, Campylobacter* if not mentioned)
4. Have you or has anyone in your household ever had foodborne illness? What symptoms made you/ would make you think that you or a member of your household had foodborne illness? Has anyone ever had to go to the doctor?
5. How do you get information about safe food handling?

Closing question: All things considered, what do you think the most important factor is in the prevention of food poisoning? (5 minutes)

SUMMARIZE

Ask the participants if we've missed anything or if they have any questions. Thank participants for their time. (5 minutes)
